# TLR Stimulation Dynamically Regulates Heme and Iron Export Gene Expression in Macrophages

**DOI:** 10.1155/2016/4039038

**Published:** 2016-02-24

**Authors:** Mary Philip, Edison Y. Chiu, Adeline M. Hajjar, Janis L. Abkowitz

**Affiliations:** ^1^Division of Hematology, University of Washington, Seattle, WA 98195, USA; ^2^Department of Comparative Medicine, University of Washington, Seattle, WA 98195, USA

## Abstract

Pathogenic bacteria have evolved multiple mechanisms to capture iron or iron-containing heme from host tissues or blood. In response, organisms have developed defense mechanisms to keep iron from pathogens. Very little of the body's iron store is available as free heme; rather nearly all body iron is complexed with heme or other proteins. The feline leukemia virus, subgroup C (FeLV-C) receptor, FLVCR, exports heme from cells. It was unknown whether FLVCR regulates heme-iron availability after infection, but given that other heme regulatory proteins are upregulated in macrophages in response to bacterial infection, we hypothesized that macrophages dynamically regulate FLVCR. We stimulated murine primary macrophages or macrophage cell lines with LPS and found that* Flvcr* is rapidly downregulated in a TLR4/MD2-dependent manner; TLR1/2 and TLR3 stimulation also decreased* Flvcr *expression. We identified several candidate TLR-activated transcription factors that can bind to the* Flvcr* promoter. Macrophages must balance the need to sequester iron from systemic circulating or intracellular pathogens with the macrophage requirement for heme and iron to produce reactive oxygen species. Our findings underscore the complexity of this regulation and point to a new role for FLVCR and heme export in macrophages responses to infection and inflammation.

## 1. Introduction

Nearly all organisms require iron because of its ability to catalyze redox reactions, and humans have evolved mechanisms to recycle almost all the iron contained within hemoglobin and cellular enzymes with only minimal daily loss through the gastrointestinal tract [1]. Nevertheless, iron-deficiency anemia and anemia of chronic disease (caused in part by iron-restricted erythropoiesis) together are responsible for the majority of anemia cases worldwide [2], and while these conditions cause morbidity and mortality, it has become clear that limiting iron availability is in fact an innate immune strategy against microbes. Indeed studies in humans and mice have shown that oral iron supplementation leads to increased mortality due to infection [3, 4]. Pathogens that enter and proliferate within a host must acquire iron from the host and have evolved a large and diverse number of mechanisms to accomplish this [5], and in response, mammals have developed complex mechanisms to keep iron from pathogens [6, 7].

Over the last two decades, there has been much effort aimed at understanding the pathophysiology of anemia associated with chronic inflammation/disease, characterized by hypoferremia. This led to the finding that a small peptide, hepcidin, initially identified as an antimicrobial peptide [8], is a master regulator of systemic iron stores. Hepcidin is produced mainly by the liver and its production is regulated by inflammation/iron levels, hypoxia, and erythropoiesis; hepcidin is elevated in anemia of chronic inflammation [9]. Hepcidin can bind to ferroportin 1 (FPN1), an iron export protein found on macrophages, enterocytes, and other cell types, and in doing so causes FPN1 internalization and degradation [10]. This results in sequestration of iron within macrophages and decreased intestinal absorption. The vast majority of bodily iron stores is complexed with heme in hemoglobin in red cells, and senescent red cells are broken down and phagocytosed by specialized macrophages in the spleen, which are highly efficient in recycling iron from hemoglobin. Nevertheless, pathogens have evolved complex mechanisms to obtain heme from blood or tissues as an alternate source of iron [11]. While the heme synthesis pathway has been well characterized in mammals, much less is known about how heme is regulated or trafficked within cells or systemically [12]. We identified the feline leukemia virus subgroup C receptor (FLVCR), a 12-transmembrane domain protein and member of the major facilitator superfamily, as a heme exporter in mammalian cells [13, 14]. Heme exported from cells through FLVCR is rapidly bound by plasma proteins including hemopexin and albumin, which can then transport the heme to other sites for utilization [15]. We found that FLVCR is required for normal erythroid [16] and T lymphocyte development [17]. Although macrophages express high levels of FLVCR, consistent with a role for macrophages in recycling heme/heme iron from phagocytosed senescent red cells [16], the role of FLVCR in regulating heme-iron after infection remains unexplored. Macrophages upregulate heme oxygenase-1 (HMOX1), a heme-degrading enzyme, in response to inflammation or infection [18]; therefore we hypothesized that macrophages dynamically regulate FLVCR in response to inflammation or infection.

We analyzed* Flvcr* (also referred to as* Mfsd7b*) mRNA levels as well as that of the other key heme and iron regulatory genes,* Fpn1 *(also referred to as* Slc40a1*),* Hmox1*, and* ferritin light chain* (*Ftl*) in macrophages treated with LPS. We found that* Flvcr* mRNA decreases quickly upon LPS stimulation, similar to* Fpn1*, before recovering to baseline 24–48 hours later. The return to baseline* Flvcr* expression coincides with the major increase in* Hmox1* and* Ftl1* expression, suggesting an initial need for increased heme in macrophages after infection accomplished by FLVCR downregulation, then at later time points, heme is degraded by HMOX1 and iron is sequestered in ferritin. While macrophage sequestration of heme and iron may be one aspect of antimicrobial defense, macrophages need heme and iron for the reactive oxygen (ROS) production and bacterial killing [19, 20]. These observations suggest that, upon infection, macrophages initially transiently increase intracellular heme and iron in order to kill bacteria before shifting to a strategy focused on iron sequestration.

## 2. Materials and Methods

### 2.1. Macrophage Cell Culture

The J774A.1 cell line (henceforth referred to as J774) was obtained from ATCC and maintained in cDMEM: DMEM medium (Gibco) supplemented with 10% inactivated FBS, penicillin/streptomycin/L-glutamine (1 unit/mL, 1 *μ*g/mL, and 2 mM; Gibco), HEPES (10 mM; Gibco), *β*-mercaptoethanol (0.05 M; Sigma), and MEM-non-essential amino acids (0.1 mM; Gibco). Bone-marrow-derived macrophages (BMDM) were prepared by euthanizing 6–8-week-old male C57BL/6 mice and sterilely dissecting both femurs. The bone marrow was flushed from the femur with HBSS, homogenized into single cell suspension by pipetting, and centrifuged at 400 g × 4 minutes (4°C) and supernatant aspirated. The cells were then resuspended at 5 × 10^6^ cells/mL in BMDM media: RPMI1640 (Gibco) supplemented with 20% inactivated FBS, 30% L929-conditioned media (LCM), penicillin/streptomycin/L-glutamine (1 unit/mL, 1 *μ*g/mL, 2 mM; Gibco), *β*-mercaptoethanol (0.05 M; Sigma), and 10 mL plated/10 cm sterile nontissue culture-treated Petri dish (Corning). After 4-5 days, the media containing nonadherent cells were removed and replaced with fresh media. BMDM were harvested by trypsin/versene treatment and replated at lower density as below for stimulation assays. BMDM were used 6–8 days after harvesting.

### 2.2. Mice

C57BL/6 mice were purchased from The Jackson Laboratory.* Tlr4* ^−/−^;* Ly96* ^−/−^ mice were previously described [21]. All mice were bred and maintained in a specific pathogen-free barrier facility at the University of Washington. Experiments were performed in compliance with the University of Washington Institutional Animal Care and Use Committee regulations.

### 2.3. Macrophage Stimulation

J774 or BMDM were plated at 5 × 10^5^ cells/well in 12-well tissue culture plates in cDMEM (J774) or BMDM media and allowed to adhere overnight. The following day, the media were exchanged for fresh cDMEM with varying concentrations of hemin or LPS (*E. coli* O111:B4; Sigma) for varying durations. For later experiments, including those using* Tlr4* ^−/−^;* Ly96* ^−/−^ BMDM, O111:B4 ultrapure LPS from InvivoGen was used. Pam3Csk4 was obtained from EMC Microcollections and polyinosinic:polycytidylic acid (pIC) from Amersham.

### 2.4. mRNA Isolation and Quantitative RT-PCR

At the appropriate time points, media were aspirated from stimulation wells and macrophages lysed with RLT buffer (Qiagen) and stored at −80°C. RNA was then purified from lysate using RNeasy Plus Mini Kit (Qiagen) and reverse-transcribed using iScript reverse transcriptase (BioRad). Multiplex quantitative real-time PCR (qPCR) was performed on cDNA using the KAPA ProbeFast BioRad iCycler reaction mix (KAPA Biosystems) with gene-specific primers obtained from Integrated DNA Technology (IDT). Primer sequences:  *β-actin* (F 5′-ACCTTCTACAATGAGCTGCG-3′, R 5′-CTGGATGGCTACGTACATGG-3′, 5′-/5Cy5/TCTGGGTCATCTTTTCACGGTTGGC/3IAbRQsp/-3′;* Flvcr* (F 5′-ATCTGGAACCTGTGCAGAAACA-3′, R 5′-ATTGAATAAAATGCTCCAGTCATGAT-3′, Probe 5′/HEX/CCCCTTTGTTCTCCTGCTGGTCAGTTATG/IABkFQ/-3′);* Hmox1* (F 5′-CTGCTAGCCTGGTGCAAGATACT-3′, R 5′-GTCTGGGATGAGCTAGTGCTGAT-3′, Probe 5′-/FAM/AGACACCCCGAGGGAAACCCCA/IABkFQ/-3′);* Fpn1* (F 5′-CCAACCGGAAATAAAACCACAG-3′), (R 5′-AGGAGAAAACAGGAGCAGATTAG-3′), (Probe 5′-/FAM/CCAACCGGAAATAAAACCACAG/IABkFQ/-3′); and* Ftl1* (F 5′-CAGCCATGACCTCTCAGATTC-3′), (R 5-CCACGTCATCCCGATCAAAA-3′), (Probe 5′-/HEX/CGCCTGGTCAACTTGCACCTG/IABkFQ/-3′).* Flvcr*,* Hmox1*, and *β-actin* primer sets were run together, and* Fpn1*,* Ftl1*, and *β-actin* primer sets were run together. Gene expression (mRNA RQ) was quantified as fold-change expression using the Pffafl method [22]; *β-actin* was the reference gene and untreated (0 ng/mL) cells were the reference sample. A dilution series of untreated C57BL/6 macrophage cDNA was run for every assay to determine the reaction efficiency for the Pfaffl calculation, to ensure that amplification was linear, and to ensure that the samples being assayed were within the linear range of the assay.

### 2.5. Macrophage Polarization

Polarization of BMDM to the M1 and M2 states was performed using established protocols [23] as follows. BMDM were prepared and 1 × 10^6^ BMDM plated per well of 6-well plates. The following day, either IFN*γ*/LPS (100 U/mL; 100 ng/mL), IL4 (100 U/mL), or nothing was added and cells were lysed at various times afterward as above for RNA isolation, cDNA production, and qRT-PCR. IFN*γ* and IL4 were obtained from eBioscience.

### 2.6. Transcription Factor (TF) Binding to* Flvcr* Promoter

The Promoter Binding TF Profiling Plate Array (Signosis) was used to assess TF binding to the murine* Flvcr* promoter. In brief, this 96-well plate-based competition assay utilizes an array of biotinylated oligos specific for 48 transcription factors (in duplicate). Nuclear extracts from the cell of interest are incubated with purified, PCR-amplified* Flvcr* promoter. The purified promoter competes with biotinylated TF-specific oligos for binding to TFs present in the nuclear extract. If there is no competition, each TF-bound oligo can hybridize to its specific complementary DNA on the 96-well plate and be detected through luminescence. TF that are present in the extract and bind to the* Flvcr* promoter will show decreased signal in the presence of the promoter compared to no promoter. Nuclear extracts were prepared from 1 × 10^6^ BMDM using the Nuclear Extraction Kit (Signosis), and the protein content was determined by the Bradford assay. 10 *μ*g of BMDM nuclear extract was incubated with or without 15 pmol of purified PCR-amplified murine* Flvcr* promoter, processed, and hybridized to the TF array plate following the manufacturer's protocol. Bound probe was detected by chemiluminescence.

## 3. Results

### 3.1. *Flvcr* mRNA Levels in Macrophages Do Not Change in Response to Heme

**Figure 1:**
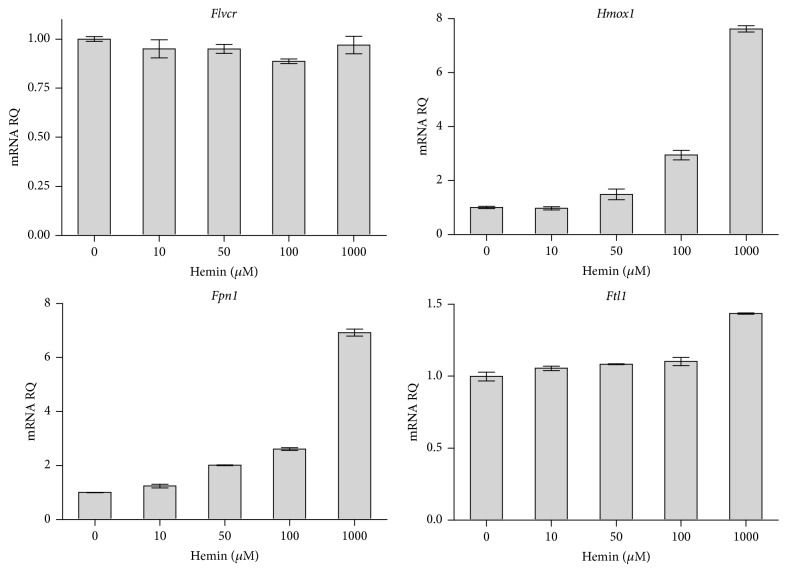
Macrophages do not regulate* Flvcr* in response to heme treatment. J774 macrophages were treated with hemin and mRNA levels of* Flvcr*,* Hmox1*,* Fpn1*, and* Ftl1* at 10 hours are shown. There was no change in macrophage* Flvcr* expression with hemin treatment, in contrast to* Hmox1*,* Fpn1*, and* Ftl1*, which increased. Similar trends were seen at 3 and 24 hours (data not shown). Expression levels are shown as mRNA relative quantity (RQ) of treated cells relative to nontreated cells and normalized to *β*-actin expression. The mean and range of duplicate samples are shown. The data is representative of 2 independent experiments.

Previously, we observed that macrophages expressed high levels of FLVCR, consistent with a role for macrophages in recycling heme/heme iron from phagocytosed senescent red cells [16]. To determine whether* Flvcr* expression in macrophages is regulated by heme, J774 macrophages were exposed to increasing doses of hemin for different times and then* Flvcr*,* Hmox1*,* Fpn1*, and* Ftl1* mRNA levels were determined by multiplex quantitative RT-PCR (qPCR). While the* Hmox1*,* Fpn1*, and* Ftl1* mRNA showed a dose-responsive increase to hemin exposure for 10 hours,* Flvcr* mRNA levels did not change (Figure 1). Similar results were seen at 3 and 24 hours (data not shown). This led us to ask whether FLVCR in macrophages might have another role aside from heme regulation after erythrophagocytosis. Macrophages are key regulators of systemic iron balance that maintain organismal iron supply while sequestering iron from pathogens [1]. Given that much of the body iron store is found in heme, we next tested whether macrophages modulate FLVCR expression in response to infection.

### 3.2. Macrophages Downregulate* Flvcr* Expression in Response to LPS Stimulation

**Figure 2:**
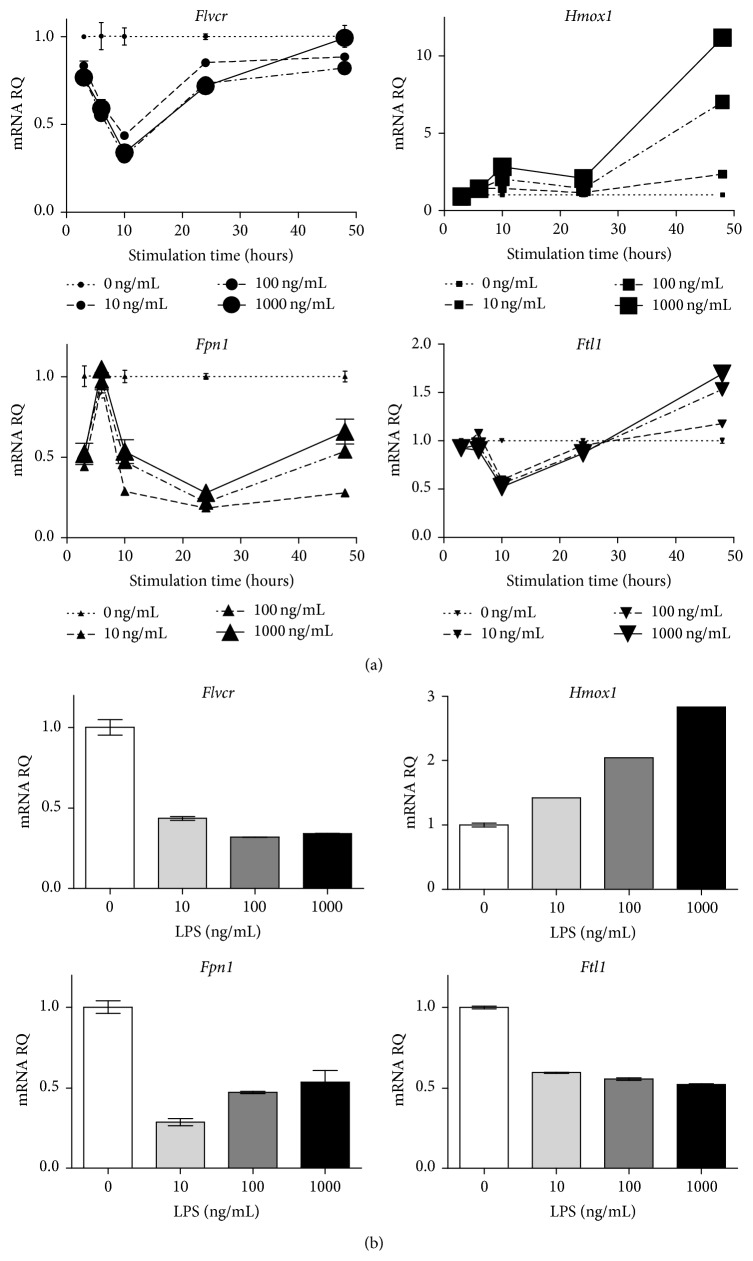
Macrophages downregulate* Flvcr* in response to LPS stimulation. (a) J774 cells were stimulated with LPS at varying doses and times. Multiplex qPCR was then performed to assess mRNA levels of* Flvcr*,* Fpn1*,* Hmox1*, and* Ftl1*. The maximal decrease in* Flvcr* expression occurred at 10 hours and then recovered to baseline. (b) mRNA levels from the 10-hour time point in (a) are shown as bar graphs to demonstrate that* Flvcr* downregulation was LPS dose-responsive. Expression levels are shown as mRNA relative quantity (RQ) of LPS-treated cells relative to nontreated cells and normalized to *β*-actin expression. The mean and range of duplicate samples are shown. The data is representative of 3 independent experiments.

*Fpn1* transcription is downregulated in macrophages stimulated by LPS [24, 25]; therefore we stimulated J774 with varying concentrations of LPS for different durations and quantified mRNA levels of* Flvcr* and key heme/iron regulatory genes. We found that* Flvcr* expression decreased rapidly upon LPS stimulation before recovering to baseline at 24–48 hours (Figure 2(a)).* Fpn1* increased rapidly and transiently before then decreasing over the first 24 hours;* Fpn1* recovery was slower and not complete by 48 hours (Figure 2(a)). This transient increase in* Fpn1* expression prior to downregulation has not been previously described. As expected,* Hmox1* expression increased with time.* Ftl1* kinetics were similar to those of* Flvcr* though the initial decrease in expression was not as marked as* Flvcr* and at later time points* Ftl1* increased above baseline (Figure 2(a)).

The decrease in* Flvcr* expression was dose-responsive between 0 and 100 ng with no further decrease at higher LPS doses, as seen in Figure 2(b), which shows heme and iron regulatory gene expression at 10 hours. Both* Flvcr* and* Hmox1* show a dose-responsive decrease and increase in mRNA expression, respectively, whereas* Fpn1* and* Ftl1* had the maximal drop in expression at the lowest dose of LPS, 10 ng/mL (Figure 2(b)).

**Figure 3:**
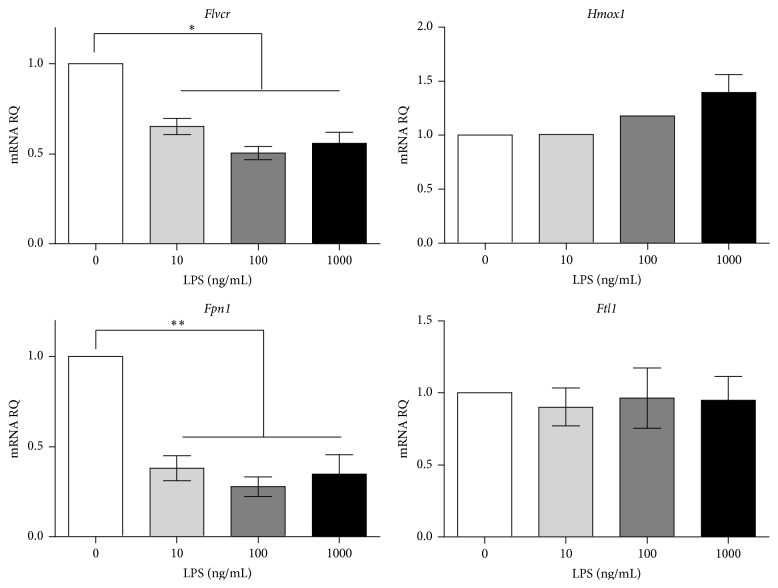
Primary bone-marrow-derived murine macrophages (BMDM) downregulate* Flvcr* in response to LPS stimulation. Primary macrophages were treated with varying doses and duration of LPS and showed a dose-responsive decrease in both* Flvcr* and* Fpn1* levels in response to 24 hours of LPS treatment. The mean and SEM are shown (*n* = 3 mice). The data is representative of 3 independent experiments. ^*∗*^ *p* < 0.001. ^*∗∗*^ *p* < 0.003.

We next assessed the effect of LPS stimulation on primary murine bone-marrow-derived macrophages (BMDM) and again observed a dose-responsive decrease in* Flvcr* similar to the decrease in* Fpn1* expression (Figure 3). The kinetics of* Flvcr* and* Fpn1* downregulation in response to LPS were similar to that seen in J774 (data not shown). Thus, both primary BMDM and macrophage cell lines respond to LPS signaling by downregulating heme and iron export.

### 3.3. *Flvcr* Expression is Differentially Regulated by Macrophage Polarization

**Figure 4:**
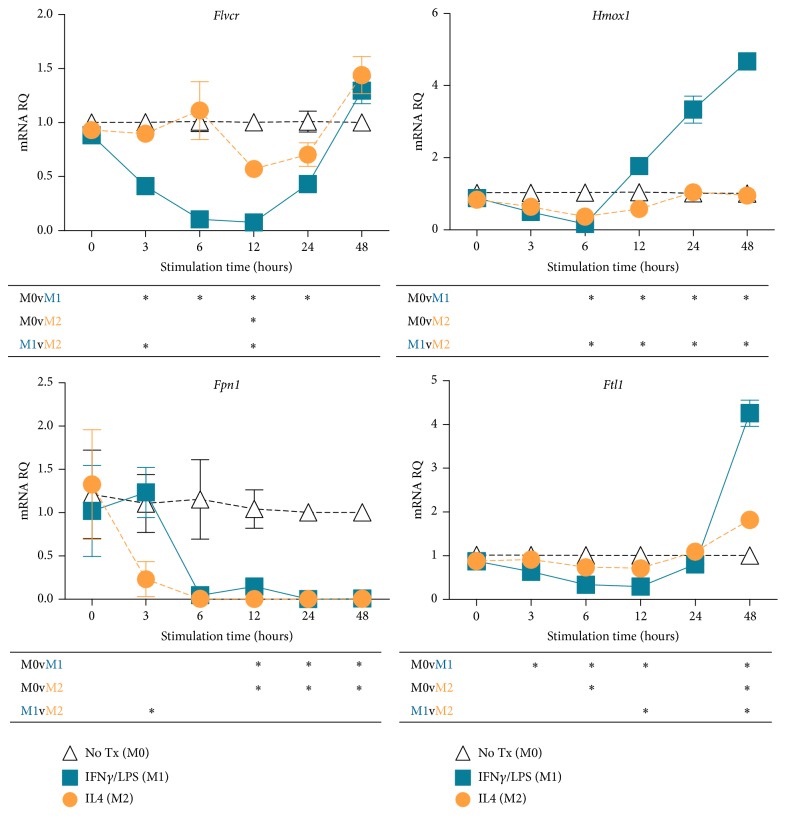
Macrophages downregulate* Flvcr *and* Fpn1* expression in response to M1 and M2 polarization. Primary macrophages (M0) were treated with LPS/IFN*γ* or IL4 to polarize to M1 or M2 state, respectively. Both M1- and M2-polarized macrophages downregulated* Fpn1* to a similar extent as compared to M0 macrophages. Interestingly,* Flvcr* downregulation in M2 macrophages occurred later and was less pronounced than in M1 macrophages. The mean and SEM are shown (*n* = 3 mice). Below each graph is a table summarizing statistical significance for all pairwise comparisons at each time point. *∗* indicates *p* ≤ 0.05. The data is representative of 2 independent experiments.

Activation of macrophages* in vitro* or* in vivo* by various stimuli leads to distinct gene expression patterns, a process referred to as macrophage polarization [23]. LPS and IFN*γ* treatment leads to M1 macrophage polarization, suited for combating infection and acute inflammation, whereas IL4 treatment leads to M2 polarization. M2 macrophages promote tissue regeneration and the return to baseline homeostasis. Most studies on macrophage regulation of iron balance have been done on M1 macrophages, but one study found that in contrast to M1 macrophages, M2-polarized human macrophages do not sequester iron but rather release iron to the surrounding tissues, promoting proliferation [26]. To determine whether* Flvcr* was differentially regulated in M1- versus M2-polarized macrophages, we generated primary murine undifferentiated macrophages (M0) and then treated with either LPS and IFN*γ* or IL4 to generate M1- and M2-polarized macrophages. Similar to previous reports, we found that* Hmox1* and* Ftl1* expression was not upregulated in M2 macrophages (Figure 4). However, both M1 and M2 macrophages downregulated* Fpn1* mRNA, and in contrast to LPS treatment alone,* Fpn1* mRNA remained suppressed at 48 hours. Interestingly,* Flvcr* expression decreased later and to a much lower extent in M2 versus M1 macrophages. Thus, M2 macrophages maintain* Flvcr* expression, possibly to export heme to cells in regenerating tissues.

### 3.4. LPS-Induced* Flvcr* Downregulation Is TLR4-Dependent

**Figure 5:**
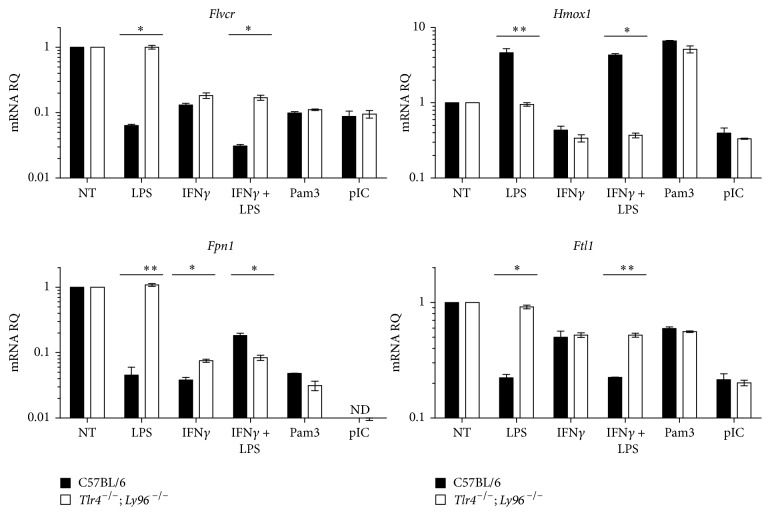
Macrophages downregulate* Flvcr* expression in response to multiple TLR agonists, and the downregulation is TLR-dependent. Primary BMDM were generated from control C57BL/6 or* Tlr4* ^−/−^ and* Ly96* ^−/−^ mice and stimulated with various TLR agonists and IFN*γ*.* Flvcr* and* Fpn1* downregulation in response to LPS was abrogated in* Tlr4* ^−/−^ and* Ly96* ^−/−^ macrophages. Stimulation with TLR1/2 agonist Pam3Csk4 (Pam3) and the TLR3 agonist polyinosinic:polycytidylic acid (pIC) also caused a decrease in* Flvcr* and* Fpn1* expression in both wild-type and* Tlr4* ^−/−^;* Ly96* ^−/−^ macrophages. The mean and SEM are shown (*n* = 3 mice). ND: not detected, as amplification was below the lower limit of detection. The data are representative of 2 independent experiments. ^*∗*^ *p* ≤ 0.01. ^*∗∗*^ *p* ≤ 0.001.

LPS binds to the TLR4 receptor complex on macrophages to activate multiple downstream signaling pathways [27]. To confirm that LPS-induced* Flvcr* downregulation was mediated by the TLR4 pathway, we stimulated BMDM from* Tlr4* ^−/−^;* Ly96* ^−/−^ mice or controls with LPS or LPS/IFN*γ*.* Ly96* encodes MD2, a coreceptor with TLR4 required for LPS signaling. Loss of TLR4 and MD2 completely reversed LPS-induced downregulation of* Flvcr* and* Fpn1* (Figure 5). To demonstrate specificity, we stimulated wild-type and* Tlr4* ^−/−^;* Ly96* ^−/−^ BMDM with the TLR1/2 agonist Pam3Csk4 (Pam3) and TLR3 agonist polyinosinic:polycytidylic acid (pIC). We found that both Pam3 and pIC treatment led to* Flvcr* and* Fpn1* downregulation in both wild-type and* Tlr4* ^−/−^;* Ly96* ^−/−^ BMDM (Figure 5). Another group recently showed that TLR2 and TLR6 agonists cause* Fpn1* mRNA downregulation in macrophages [28]. Thus, decreased transcription of the genes encoding both heme and iron export proteins is a common pathway following TLR activation in macrophages. Interestingly,* Hmox1* was upregulated by LPS and Pam3, but not by pIC (Figure 5), suggesting distinct regulation of* Flvcr* as compared to other heme regulatory genes.

### 3.5. Transcription Factors Activated by LPS/TLR Signaling Bind to the* Flvcr* Promoter

**Figure 6:**
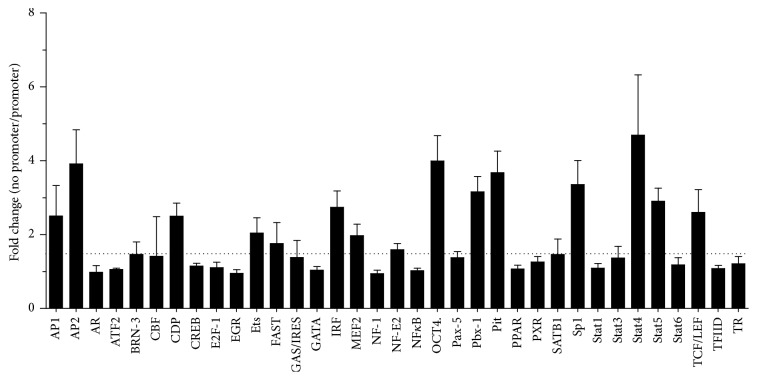
LPS-activated transcription factors in macrophages bind to* Flvcr* promoter. Nuclear extracts were prepared from primary murine BMDM and incubated with or without purified* Flvcr* promoter DNA. Transcription factor (TF) activity was measured using the Signosis Promoter Binding Transcription Factor Profiling Array 1 and results are presented as the amount of TF activity without promoter over TF activity with promoter, reflecting promoter binding. The dotted line indicates 1.5-fold change. Several of the TF with highest* Flvcr* promoter binding (STAT4, AP2, SP-1, and IRF-1) had predicted binding sites with the human* Flvcr* promoter (Supplemental Figure 1), and STAT4 and IRF-1 are known downstream mediators of LPS/TLR4 signaling in macrophages. The mean and SEM are shown (*n* = 3 mice).

To explore the connection between LPS stimulation and* Flvcr* mRNA transcription, we used the EPDnew eukaryotic promoter database [29] to identify the human* Flvcr* promoter sequence and then queried the promoter sequence for transcription factor (TF) binding sites using PROMO [30, 31]. This analysis revealed many potential binding sites motifs for transcription factors known to be expressed in macrophages such as NF-*κ*B, IRF-1, and C/EBP*β* [27] (see Supplemental Figure 1 of the Supplementary Material available online at http://dx.doi.org/10.1155/2016/4039038). We next used a multiplex TF binding assay to identify TF present in murine BMDM that can bind to the murine* Flvcr* promoter. Several of the TF with highest* in vitro Flvcr* promoter binding activity (Figure 6) such as STAT4, AP2, SP-1, and IRF-1 had predicted binding sites with the human* Flvcr* promoter (Supplemental Figure 1). Moreover, STAT4 [32] and IRF-1 [33] are known downstream mediators of LPS/TLR4 signaling in macrophages.

## 4. Discussion

In this study, we found that primary and immortalized murine macrophages downregulate* Flvcr *mRNA levels upon LPS stimulation, similar to the downregulation of* Fpn1* expression previously described [24, 25]. Macrophages also downregulated* Flvcr* and* Fpn1* in response to TLR1/2 and TLR3 agonists, suggesting that heme and iron sequestration in macrophages is a general response to inflammatory/infectious stimuli. It was previously reported that M1 polarization causes human macrophages to sequester iron through FPN1 downregulation while M2 polarization leads to increased FPN1 protein expression [26]. A more recent study [34] found that* Fpn1* expression was decreased in murine M0 macrophages polarized to both the M1 and M2 states. Interestingly, this study also found that heme (in the form of RBC or free heme) polarizes M0 macrophages to the M1 state [34]. We found that, in murine macrophages, both M1 and M2 polarization caused decreased expression of* Fpn1* and* Flvcr*, though notably the decrease in* Flvcr* expression occurred later and was less marked under M2 polarizing conditions. The hypothesis is that macrophages sequester iron in response to infection (M1) and export iron and heme under M2 conditions in which tissue regeneration and proliferating cells have higher demand for heme and iron. Future studies could explore* Flvcr* and* Fpn1* expression* in vivo* under physiologic conditions of M1 (acute infection) and M2 polarization (late-stage wound healing) or investigate how tumor-associated macrophages regulate heme and iron.

Surprisingly,* Flvcr* mRNA levels in J774 macrophages did not change significantly in response to heme treatment. While free heme may not alter* Flvcr* mRNA expression in macrophages, it is possible that* Flvcr* expression may change in response to macrophage erythrophagocytosis, especially in splenic macrophages specialized to take up senescent RBC. Heme has been shown to regulate* Fpn1* transcription through binding the transcriptional factors Btb and Cnc Homology 1 (BACH1) and Nuclear Factor Erythroid 2-like (NRF2), which associate with a conserved Maf Recognition Element (MARE)/Antioxidant Response Element (ARE) in the* Fpn1* promoter [35]. We did not identify any MARE/ARE elements in the* Flvcr* promoter.

Thus, while HMOX1 and FPN1 in macrophages may respond to changes in intracellular heme and iron levels in addition to inflammatory/infectious signals, the primary role of FLVCR in macrophages may be the regulation of heme in response to infection/inflammation. It is notable that the decrease in* Flvcr* expression occurs rapidly within 10 hours after LPS stimulation, while* Fpn1* expression had an initially small increase followed by a slower decrease in expression (Figures 2(a) and 4). Whether this transient* Fpn1* increase prior to the subsequent downregulation is functionally important is not known. The different expression kinetics suggest that* Flvcr* and* Fpn1* are regulated differently. FPN1 protein expression is also regulated posttranslationally through inflammation-induced hepcidin [6]. It is not known whether FLVCR is also posttranslationally regulated, though one study using transfected cell lines found that FLVCR had a long half-life (>16 hours) [36]. It has been difficult to study the localization and regulation of FLVCR protein* in vivo* or from* ex vivo* murine cells because there is no antibody available. Once alternative strategies (such as genetic knock-in of epitope tag sequences) make* in vivo* and* ex viv*o of murine FLVCR detection and localization feasible, it will be important to study the kinetics and trafficking of FLVCR and FPN1 protein after LPS treatment.

The decrease in* Flvcr* expression precedes the major increase in* Hmox1* expression, at which point* Flvcr* expression has returned to baseline. This suggests that the decrease in FLVCR is functionally important in the first hours after infection, and as other mechanisms for systemic iron regulation (hepcidin-mediated FPN1 degradation, increased Ferritin expression) are initiated, FLVCR returns to baseline. One explanation is that early FLVCR downregulation might be important for macrophage killing of intracellular pathogens. Heme and iron-containing enzymes produce the reactive oxygen species and other compounds needed for intracellular killing of bacteria [19]. Macrophages that have just encountered and/or endocytosed bacteria may downregulate FLVCR in order to increase heme available for cytolytic enzymes. A recent review highlighted the “macrophage paradox,” that is the finding that many pathogens preferentially replicate inside macrophages in spite of their specialized killing function [37], and different bacteria have different intracellular niches within macrophages. Given that heme and iron are also trafficked and regulated differently in intracellular compartments [12, 38–40], there are likely several layers of regulation of heme and iron regulatory proteins at transcriptional, posttranscriptional, translational, and posttranslational (including trafficking) levels required to meet the challenges presented by specific pathogens. This is supported by the recent finding that the survival of* Listeria monocytogenes *localized to different intracellular compartments in macrophages was differentially altered by FPN1 expression [41]. Moreover, macrophages are not a uniform population, but rather there are several subtypes and macrophage differentiation states which serve specific functions in different tissues and depending on the conditions [23]. Macrophages that are specialized for erythrophagocytosis may regulate heme and iron regulatory proteins in response to heme and iron levels [40] rather than inflammatory signals as we observed here. Our finding that macrophages dynamically regulate* Flvcr* expression in response to TLR signaling points to a new potential role for FLVCR and heme export in macrophages during infection and inflammation. Future studies aimed at elucidating the transcriptional and posttranscriptional regulation of FLVCR in response to TLR and inflammatory signaling will improve our understanding of the complex interplay cell and tissue-localized demands for heme and iron and systemic heme/iron homeostasis.

## Supplementary Material

Supplemental Figure 1: The EPDnew (1) eukaryotic promoter database was used to identify the *Flvcr* promoter sequence from -499 to +100 base pairs relative to the transcription start site. This sequence was queried for human transcription factor (TF) binding sites using PROMO (2) and the resulting output is shown above. Of the TF binding sites identified, many are known to be expressed in macrophages and activated by LPS signaling.
