# Supplementary material for: TLR Stimulation Dynamically Regulates Heme and Iron Export Gene Expression in Macrophages
Source: J Immunol Res. 2016 Feb 24;2016:4039038. doi: 10.1155/2016/4039038 (PMC4783552; doi:10.1155/2016/4039038)
Supplement: Supplementary file 1 — Supplemental Figure 1: The EPDnew (1) eukaryotic promoter database was used to identify the Flvcr promoter sequence from -499 to +100 base pairs relative to the transcription start site. This sequence was queried for human transcription factor (TF) binding sites using PROMO (2) and the resulting output is shown above. Of the TF binding sites identified, many are known to be expressed in macrophages and activated by LPS signaling. [file 4039038.f1.pdf]

Supplemental Figure 1

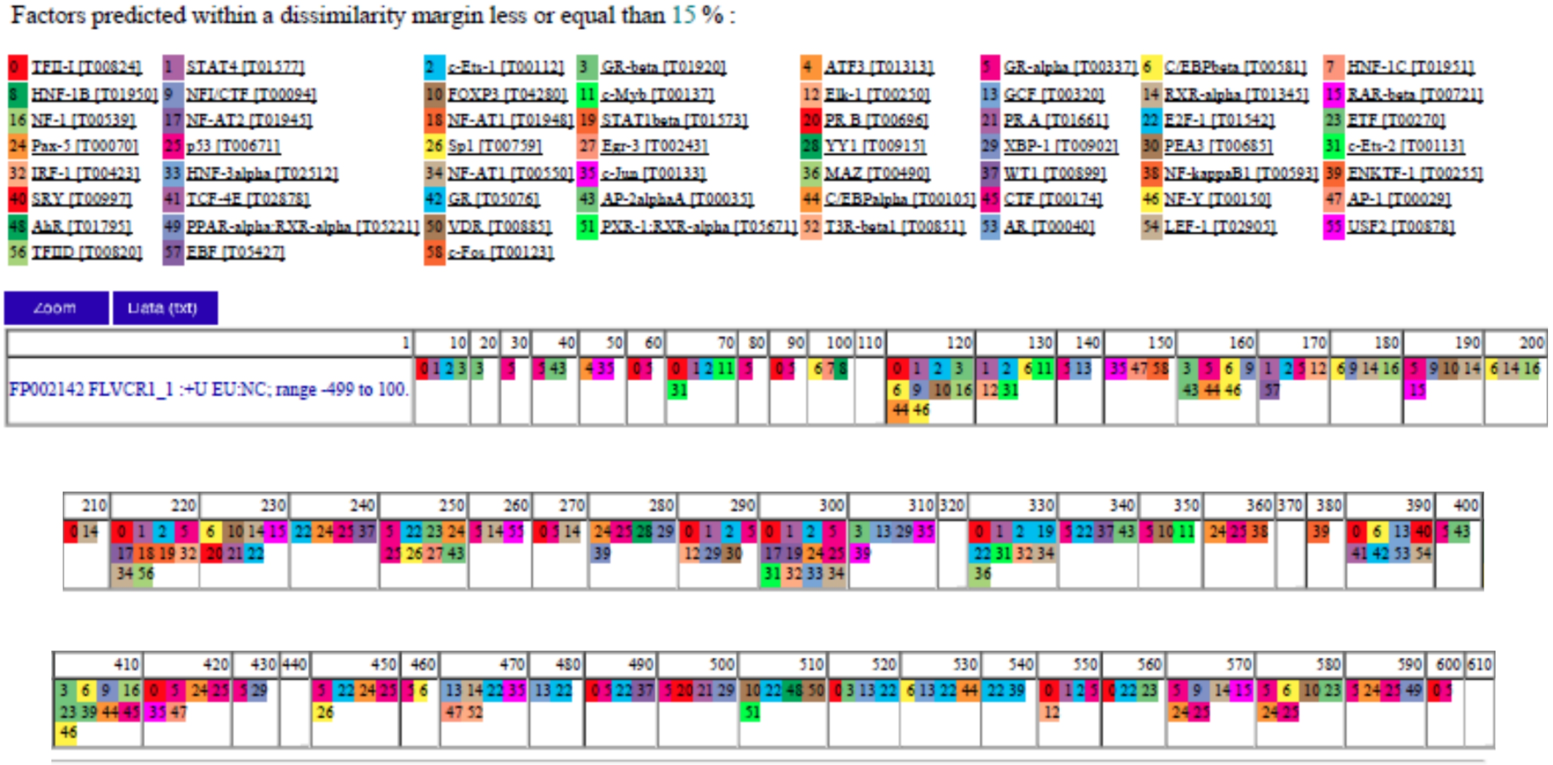

Supplemental FIG 1 The EPDnew (1) eukaryotic promoter database was used to identify the *Flvcr* promoter sequence from -499 to +100 base pairs relative to the transcription start site. This sequence was queried for human transcription factor (TF) binding sites using PROMO (2) and the resulting output is shown above. Of the TF binding sites identified, many are known to be expressed in macrophages and activated by LPS signaling.
